# Gender and sexuality in mental health: perspectives on lesbians, gays, bisexuals, and transgender (LGBT) rights and mental health in the ASEAN region

**DOI:** 10.3389/fsoc.2023.1174488

**Published:** 2023-04-27

**Authors:** Rowalt Alibudbud

**Affiliations:** Department of Sociology and Behavioral Sciences, De La Salle University, Manila City, Philippines

**Keywords:** sexual and gender minorities, mental health, Southeast Asia, gender identity, marriage equality, discrimination, LGBT rights, homosexuality

## Abstract

This perspective piece focuses on and analyzes several lesbians, gays, bisexuals, and transgender (LGBT) individuals' rights and their limitations in the Association of Southeast Asian Nations (ASEAN) region, including the limited recognition of self-determined gender identity, limited legal provisions for LGBT marriage, inadequate anti-discrimination policies, and the criminalization of homosexuality. These inadequacies in LGBT rights may stem from colonial, religious, and cultural factors. Moreover, these limited LGBT rights and their societal repercussions may contribute to the minority stress of LGBT individuals, leading to their higher rates of mental health problems. Thus, there may be a need to uphold, recognize, and protect LGBT rights as the region pursue equitable mental health. Toward this pursuit, the region may possibly benefit from culturally adapting gender-affirming practices, increasing their social support, opposing the practice of conversion therapy, and decriminalizing homosexuality. It may also be necessary to explore, analyze, and study the intersection of LGBT identity and mental health, especially longitudinal and interventional studies.

## 1. Introduction

The Association of Southeast Asian Nations (ASEAN) comprises ten member states, including Brunei Darussalam, Cambodia, Indonesia, Lao PDR, Malaysia, Myanmar, Philippines, Singapore, Thailand, and Vietnam. The ASEAN is also a region with diverse cultures, traditions, and policies (Global Health Action, 2015). Notably, at least a quarter of the population of several ASEAN member states, including Indonesia, Malaysia, the Philippines, Singapore, Thailand, and Vietnam, may have negative attitudes toward lesbians and gay men (Manalastas et al., 2017). Similarly, bisexual and transgender individuals have also reported multiple instances of discrimination in the region (UNDP and USAID, 2014; Kwatra, 2017; Langlois et al., 2017; Weiss, 2021). These attitudes may stem from colonial, religious, and cultural factors (Manalastas et al., 2017; Alibudbud, 2021; Tan and Saw, 2022). Unfortunately, legislation protecting them from these attitudes and discrimination remains sparse in the region (Kwatra, 2017; Langlois et al., 2017; Weiss, 2021).

## 2. Minority stress and sexual citizenship

Meyer's minority stress model identifies these negative attitudes and discrimination against lesbians, gays, bisexuals, and transgender (LGBT) individuals as distal stressors, which are the “external, objective stressful events and conditions (Meyer, 2003)”. As contributors to minority stress, these adverse behaviors and limited protective policies for LGBT individuals may contribute to their higher rates of mental disorders than the general population (Meyer, 2003; UNDP and USAID, 2014; Alibudbud, 2021; Tan and Saw, 2022). These mental health disparities are already evident among LGBT individuals in the ASEAN region. For example, Tan and Saw (2022) noted in their review that there is an increased prevalence of depression, non-suicidal self-injury, suicidal ideation, and suicide attempt among LGBT individuals compared to their cisgender and heterosexual counterparts in the region. Thus, it is necessary to analyze the existing rights of LGBT individuals and their possible influence on LGBT mental health.

This perspective piece reviews the legislation for several LGBT rights, their limitations, and their possible implications for mental health in the ASEAN region. While LGBT rights have been examined using various theoretical and methodological approaches and frameworks (e.g., gay and lesbian studies, queer studies) (Kuriakose and Iyer, 2020), this perspectives piece positions LGBT rights using the sexual citizenship framework of Richardson (2000). This framework is among the predominant frameworks for analyzing the right claims for LGBT people (Richardson, 2015). Its use has prompted legislative and social changes (e.g., the right to equal marriage) for LGBT people in many regions of the world (Richardson, 2015). Therefore, in the hopes of achieving similar social changes for LGBT people in the ASEAN region (Richardson, 2000), this perspective piece followed the sexual citizenship framework. In doing so, it situates LGBT rights based on discourses about conduct-based, identity-based, and relationship-based rights claims. Conduct-based discourses center on the right to sexual practice, including the participation and enjoyment of sexual acts and those concerned with “bodily self-control (Richardson, 2000).” This right concerns discriminatory legislation penalizing certain sexual practices, such as criminalizing homosexuality (Richardson, 2000). Identity-based discourses focus on the right to self-definition, self-expression, and self-realization, including the recognition of self-determined gender identity and freedom from sexual orientation, gender identity, and expression (SOGIE) based discrimination (Richardson, 2000). Lastly, relationship-based discourses emphasize the right to the public validation of diverse forms of sexual interactions, including the recognition of marriage among LGBT individuals (Richardson, 2000).

Given this framework for LGBT rights, this perspective piece focuses on, analyzes, and critiques the state of gender identity recognition and affirmation, LGBT marriage, discrimination, and the criminalization of homosexuality among ASEAN countries. Likewise, their possible roots and implications for the mental health of LGBT individuals were also explored in light of the minority stress model.

## 3. Discussion

### 3.1. Gender identity recognition and affirmation

Gender identity signifies a “person's deeply felt, internal and individual experience of gender, which may or may not correspond to the person's physiology or designated sex at birth (World Health Organization, n.d.).” Richardson (2000) identifies that the right to self-definition may entail validating and recognizing self-determined gender identity. However, the region's recognition of diverse gender identities, such as transgender and non-binary identities, is limited. For instance, Thailand does not legally recognize self-determined gender identity despite performing the highest number of sex reassignment surgeries worldwide (Kwatra, 2017). This limited recognition can be partly attributed to terms in the Marriage Condition and Family Sections of Thailand's Civil and Commercial Code, which limits recognition to an individual's sex at birth (Kwatra, 2017; Newman et al., 2021). Only Vietnam and Singapore recognize self-determined gender identity in the ASEAN region. However, gender identity is only recognized after individuals have undergone sex reassignment surgery (Kwatra, 2017).

This limited gender identity recognition may contribute to minority stress, leading to heightened rates of mental disorders among LGBT individuals in the region (Tan and Saw, 2022). A systematic review by Tan and Saw (2022) found that LGBT mental health may benefit from affirming one's gender identity. However, they also highlighted that further studies, including longitudinal and interventional research, are necessary to establish the mental health outcomes of gender affirmation Tan and Saw (2022). Nonetheless, evidence suggests that legal gender recognition without impediments, such as removing the requirement of sex reassignment surgery or hormonal therapy, may be provided to possibly improve mental health outcomes among LGBT individuals (Newman et al., 2021; Tan and Saw, 2022).

In general, LGBT individuals in the region may benefit from gender identity affirmation. In addition, the region can also culturally adapt other countries' gender-affirming guidance to fit their context. For instance, US gender-affirming practices such as gender-specific group therapy can be adapted to the Philippines by implementing this therapy among its closely-bonded peer groups called “bakadahan” (Alibudbud, 2021; American Psychiatric Association, n.d.). Nonetheless, further research, especially longitudinal studies, is needed to ascertain the benefits of gender affirmation and surgery on mental health in the region.

### 3.2. LGBT marriage and unions

Like gender affirmation, marriage may also offer protection against poor mental health (Wight et al., 2013). In addition, evidence suggests that married individuals have higher social support, intimacy, and self-worth, contributing to higher mental well-being (Wight et al., 2013). This protective effect has also been found among LGBT couples (Wight et al., 2013). Likewise, marriage may be vital in Asian cultures, where their closely-knitted communities offer readily available social support (Manalastas et al., 2017; Alibudbud, 2021; Tan and Saw, 2022). Thus, as an LGBT right, the public recognition of LGBT marriage may be important for the mental health of LGBT individuals in the ASEAN region. However, the region has limited legal provisions for LGBT marriage or unions. As a result, the additional protection afforded by marriage and romantic relationships may not extend to LGBT individuals (Kwatra, 2017; Weiss, 2021).

This limited marriage equality may be attributed to social and legal impediments. For instance, the Philippines' Family Code, similar to Thailand, states that marriage is between a “man” and a “woman” (UNDP and USAID, 2014). Without repealing this code, it may limit the eventual recognition of LGBT marriage. Often in the region, arguments against marriage equality legislation to recognize LGBT marriages centered on its “religious immorality (e.g., Islam in Indonesia and Roman Catholicism in the Philippines) (UNDP and USAID, 2014; Manalastas et al., 2017; Alibudbud, 2021, 2022a; Weiss, 2021).” A popular Philippine example of this religion-based argument was a lawmaker's claim that people who engage in same-sex intercourse are “worse than animals (Alibudbud, 2021).” Thus, LGBT couples may be deprived of higher social support afforded to married heterosexual individuals. Moreover, relationships between LGBT individuals may be distressing since they may conceal their relationships because they may be discriminated against and penalized (UNDP and USAID, 2014; Pachankis et al., 2020; Alibudbud, 2021). Hence, limited marriage equality may also contribute to minority stress since it may entail arguments surfacing public prejudice toward LGBT individuals. Since marriage may possibly benefit mental health, efforts toward marriage equality and legal unions in the region can be replicated and started. For instance, Vietnam's LGBT community celebrated the removal of their government's ban on LGBT marriage (Kwatra, 2017). This ban removal can also be reiterated and replicated among the region's nations. While awaiting marriage equality legislation, social support can also be strengthened by supporting LGBT organizations, incentivizing companies with LGBT-friendly policies, and expanding gender mainstreaming activities that include LGBT identities.

### 3.3. LGBT discrimination and anti-discrimination laws

Freedom from discrimination is an inherent right accorded to individuals. The sexual citizenship framework identifies protection from SOGIE-based discrimination as a possibly fundamental sexual right of LGBT individuals (Richardson, 2000). However, evidence suggests that negative attitudes and discrimination against LGBT individuals may be higher in several ASEAN countries than in some of their western counterparts (Manalastas et al., 2017; Tan and Saw, 2022). LGBT individuals have also reported that discrimination can occur in various settings, including homes, schools, and workplaces (UNDP and USAID, 2014; Kwatra, 2017). Likewise, they also noted that discrimination could be committed by their peers, family members, and government officials (UNDP and USAID, 2014; Kwatra, 2017; Weiss, 2021; Alibudbud, 2022a). For instance, young LGBT individuals in the Philippines have been mocked and bullied due to their gender identities by their classmates at school (UNDP and USAID, 2014). Likewise, those from Muslim families have also reported being disowned by their families due to their sexual orientation (UNDP and USAID, 2014). Outright physical and sexual abuses have also been reported in the region (UNDP and USAID, 2014). Among 25 reviewed studies in the region, it was found that these distal minority stressors consistently associate with adverse mental health outcomes, including heightened rates of depression and suicide attempts among LGBT individuals (Tan and Saw, 2022).

These negative societal attitudes and norms toward LGBT individuals are influenced by traditional religious values, especially in Malaysia, Indonesia, the Philippines, and Brunei (Manalastas et al., 2017; Tan and Saw, 2022; UNDP and USAID). For instance, Catholic values in the Philippines may promote cis-heterosexism, a view that cisgenderism and heterosexuality are society's “normal” configuration (Meyer, 2003; Alibudbud, 2021, 2022a; Tan and Saw, 2022; UNDP and USAID). This view may lead to a binary perception of gender and sexual orientation that is limited to the social classification of individuals as “men” and “women,” thereby marginalizing LGBT individuals (Meyer, 2003; Alibudbud, 2021, 2022a; Tan and Saw, 2022; UNDP and USAID).

In recent years, there have been steps toward decreasing these stressors. Thailand and some Philippine localities have policies that protect from sexuality and gender-based discrimination (Kwatra, 2017; Weiss, 2021). However, their policy implementation may be poor, largely symbolic, and limitedly applied (Kwatra, 2017; Weiss, 2021). For instance, the national law in Thailand has several exemptions, which may decrease its effectiveness in mitigating discrimination (Kwatra, 2017). Hence, LGBT individuals may remain oppressed and abused. To cope with adversities, they conceal their gender identities and sexual orientation. Identity concealment may also be detrimental to mental health (Meyer, 2003; Pachankis et al., 2020; Alibudbud, 2021; Tan and Saw, 2022; UNDP and USAID). Thus, LGBT individuals may remain highly distressed whether they hide or live their sexual and gender identities.

Since discrimination may be detrimental to LGBT mental health, anti-discrimination policies may need to be strengthened (Meyer, 2003; Alibudbud, 2021; Tan and Saw, 2022; UNDP and USAID). Toward these policies, dialogues about gender inclusivity between lawmakers and the LGBT community, as seen in Thailand and Vietnam, may be increased and replicated in the region (UNDP and USAID, 2014; Kwatra, 2017; Tan and Saw, 2022). Furthermore, since psychiatry has a role in promulgating the pervasive LGBT stigma, psychiatrists may join the societal call and movement for LGBT rights in the region (The Lancet Psychiatry, 2022). For instance, similar to the Indian Psychiatric Association and Taiwan Society of Psychiatry, psychiatric associations in the ASEAN region may denounce the use of Conversion therapy and the medicalization of homosexuality (Focus Taiwan, 2017; Indian Psychiatric Association, 2020)). Principles for supporting LGBT individuals from other countries may also be adapted. For instance, the guidance of the Australian Capital Territory and the US' best practices for gender-affirming mental healthcare, such as using preferred names and pronouns, can be easily adapted in the region (Australian Capital Territory, 2021; American Psychiatric Association, n.d.).

### 3.4. Criminalization of homosexuality

The participation and enjoyment of sexual acts and autonomy over one's body were important aspects of sexual rights based on conduct-based discourses (Richardson, 2000). Among others, these rights emphasize the freedom from discriminatory policies that penalize particular sexual behaviors (Richardson, 2000). The oppression of this right is arguably epitomized in state policies that criminalize homosexuality in the ASEAN region. For example, some countries in the region, such as Malaysia and Myanmar have, penal sanctions that outlaw “unnatural offenses” and criminalize sexual activities against the “natural order (Kwatra, 2017; Human Rights Watch, 2022).” These penalties are also performed in Aceh, Indonesia, where a 2009 law penalizes homosexuality with lashes of cane and imprisonment (Kwatra, 2017; Weiss, 2021). In Brunei, the penalty is more severe, where homosexuality is punished with death penalty (Kwatra, 2017). Many of these laws reflect their “British colonial past,” where they have iterations of the Indian Penal Code, which criminalize “sexual activities against the order of nature (Kwatra, 2017; Langlois et al., 2017; Weiss, 2021)”. Likewise, these laws reflect their religious values, such as in the predominantly Muslim country of Brunei and the region of Aceh, Indonesia (UNDP and USAID, 2014; Kwatra, 2017; Weiss, 2021; Tan and Saw, 2022).

While these penalties are rarely enforced, they may perpetuate the societal stigma against LGBT individuals in the region (Kwatra, 2017). In addition, these laws may also be sources of minority stress, where LGBT individuals are exposed to substantial fears of penalties and even death for their gender and sexual identities. As a result, they may hide their identities to cope with fear and penalties (UNDP and USAID, 2014; Kwatra, 2017; Tan and Saw, 2022). Fear and concealment of identity are both highly distressing experiences, furthering minority stress among LGBT individuals. Thus, decriminalizing homosexuality may be necessary to decrease minority stress (Meyer, 2003; UNDP and USAID, 2014; Kwatra, 2017; Pachankis et al., 2020; Alibudbud, 2021; Tan and Saw, 2022).

Recently, there has been a movement in the region toward ending the criminalization of homosexuality. For example, in 2022, LGBT individuals in Singapore celebrated their government's move to abolish the colonial-era 377A law, which criminalizes sex between men (Alibudbud, 2022b; Wong, 2022). The Singapore government's reason for the move was the friendlier and more accepting public attitudes toward LGBT individuals (Alibudbud, 2022b; Wong, 2022). Thus, other countries in the region may also reassess their citizens' attitudes to guide them toward gender-inclusive policies.

## 4. Conclusion

This perspective piece identified several LGBT rights in the ASEAN region following the sexual citizenship framework of Richardson (2000), including limited recognition of self-determined gender identity, limited legal provisions for LGBT marriage, inadequate anti-discrimination policies, and the criminalization of homosexuality. It described the possible repercussions of these rights and their limitations to mental health. Using the minority stress model of Meyer (2003), the continued inadequacies in LGBT rights and their repercussions in the region may contribute to the higher rates of mental health problems among LGBT individuals in the region. Thus, it may be necessary to uphold, recognize, and protect LGBT rights as the region pursue equitable mental health. As a start, these may be addressed by culturally adapting gender-affirming practices, increasing their social support, opposing the practice of Conversion therapy, and decriminalizing homosexuality. Another challenge facing the region is the dearth of research about the intersection of gender, sexuality, and mental health, especially longitudinal and interventional studies (Tan and Saw, 2022). Thus, research on LGBT-specific mental health needs may also be strengthened, including gender affirmation, gender dysphoria, and sex reassignment surgery. Moreover, LGBT rights and mental health can be further analyzed using other conceptual and methodological frameworks and approaches (e.g., intersectionality as an analytical framework) to understand, critique, and advance sexuality, gender, and mental health discourse and scholarship in the region, especially as regional policies develop.

## Data availability statement

The original contributions presented in the study are included in the article/supplementary material, further inquiries can be directed to the corresponding author.

## Author contributions

RA had substantial contributions to the design, drafting, revision, acquisition, interpretation, and final approval of the data and work.
